# Maternal education-related inequality in female genital mutilation/cutting among daughters aged 0–14 years in three East African countries: DHS data from 2016–2022

**DOI:** 10.1080/16549716.2026.2695724

**Published:** 2026-07-08

**Authors:** Tigist Kifle Tsegaw, Nigat Amsalu Adiss, Yabibal Misganaw Mesfin, Wubedle Zelalem Temesgan, Setegn Fentahun, Fetlework Gubena Arage, Eyob Akalewold Alemu

**Affiliations:** a Department of Public Health, Institute of Public Health, College of Medicine and Health Sciences, University of Gondar, Gondar, Ethiopia; b Department of Epidemiology and Biostatistics, Institute of Public Health, College of Medicine and Health Sciences, University of Gondar, Gondar, Ethiopia; c College of Medicine and Health Sciences, Department of Gynecology and Obstetrics, University of Gondar, Gondar, Ethiopia; d Department of Psychiatry, College of Medicine and Health Science, University of Gondar, Gondar, Ethiopia; e Department of Clinical Midwifery, School of Midwifery, College of Medicine and Health Sciences, University of Gondar, Gondar, Ethiopia; f Department of Neonatal Health Nursing, School of Nursing, College of Medicine and Health Science, University of Gondar, Gondar, Ethiopia

**Keywords:** Erreygers Concentration Index, Demographic and Health Survey, inequality, decomposition analysis, child health

## Abstract

**Background:**

Globally, more than 230 million girls and women have undergone female genital mutilation/cutting (FGM/C). It is estimated that about one-fourth of them reside in East Africa. Studies highlight educational attainment as one of the major factors contributing to the practice.

**Objective:**

This study aimed to assess maternal education-related inequality in FGM/C among daughters aged 0–14 years by using Demographic and Health Survey (DHS) data (2016–2022) from three East African countries.

**Methods:**

Stata version 17 was used for the data analysis. We analyzed pooled, nationally representative DHS data (2016–2022) from three East African countries (Ethiopia, Kenya, and Tanzania), involving a weighted sample of 23,596 daughters. Maternal education-related inequality was measured using the Erreygers Normalized Concentration Index. A decomposition analysis was conducted to determine the percentage contribution of maternal and household-level factors to the observed inequality.

**Results:**

The findings revealed a pro-low-education distribution of the practice, with an Erreygers index of −0.126 (*p* < 0.001), indicating that FGM/C is significantly concentrated among daughters of less educated mothers. Decomposition analysis showed that maternal education accounted for the largest share of the observed inequality (42.51%), followed by maternal FGM/C status (23.79%), non-exposure to media (22.58%), rural residence (13.86%), and maternal age < 18 years at first cohabitation (8.7%), whereas the wealth index narrowed the inequality by 23.44%.

**Conclusions:**

FGM/C is disproportionately concentrated among daughters of less educated mothers. The observed inequality was largely shaped by maternal factors, indicating that limited educational attainment and intergenerational transmission play a substantial role in sustaining FGM/C among daughters.

## PAPER CONTEXT

- **Main finding**: Female genital mutilation/cutting was disproportionately concentrated among daughters of mothers with lower educational attainment, and maternal female genital mutilation/cutting status played a major role in the observed inequality.
- **Added knowledge**: This study provides evidence from three East African countries using nationally representative data and inequality decomposition methods to quantify the relative contribution of maternal and household factors to the observed maternal education-related inequality in female genital mutilation/cutting among daughters aged 0–14 years.
- **Global health impact for policy and action**: To reduce inequalities and speed up the elimination of female genital mutilation/cutting, it is essential to focus on improving maternal education, delaying early marriage, expanding access to information, and strengthening child protection systems alongside the existing legal framework.

## Background

Female genital mutilation/cutting (FGM/C) is the partial or total removal of the external female genitalia, or other injury to the female genital organs, for non-medical reasons [1]. The World Health Organization (WHO) categorizes FGM/C into four types. Type 1 involves the removal of the clitoral glans; Type 2 includes the removal of the clitoral glans and labia minora; Type 3, known as infibulation, is the narrowing of the vaginal opening; and Type 4 covers all other harmful procedures such as pricking, piercing, or cutting, done for non-medical reasons [2].

FGM/C has both short-term and long-term complications for girls and women, leading to a range of physical, obstetric, sexual, and psychological consequences [3–5]. The most common immediate complications include excessive bleeding, severe pain, infection, urinary retention, and swelling of the genital tissues [6–9]. The long-term gynecological impacts are also significant; women who have undergone FGM/C are more likely to experience perineal tears, prolonged labor, and an increased need for episiotomy during childbirth, depending on the type of FGM/C performed [2,10–12]. Its impact on women’s sexual health is also profound [13,14].

Globally, more than 230 million girls and women have undergone FGM/C, with Africa representing the highest proportion at over 144 million cases; an additional 30 million girls are at risk of being cut before their 15th birthday. It is estimated that about one-fourth of the survivor girls and women are from East Africa, including Ethiopia, Kenya, Somalia, Tanzania, and Uganda [15,16]. The prevalence of FGM/C varies widely across Africa, ranging from a high of 99% in Somalia to a low of 0.3% in Uganda [17]. In 2022, the prevalence of FGM/C among women aged 15–49 remained high in several East African countries, with rates of 98% in Somalia, 88% in Sudan, 74% in Ethiopia, and 21% in Kenya [18]. A previous study identified religion and various socioeconomic factors, such as education level, literacy, wealth quintile, place of residence, and ethnicity, as key contributors to the practice [19–21].

Sustainable Development Goal (SDG) 5.3 aims to eliminate all harmful practices, such as child, early, and forced marriage, and female genital mutilation, by 2030 [22]. In support of this goal, UNFPA, in partnership with UNICEF, launched a joint program in 2008. This initiative has been implemented in collaboration with governments, national and community-based organizations, and other key stakeholders across 18 African countries to eliminate FGM/C by 2030. Since 2012, the Africa Coordinating Centre for the Abandonment of FGM/C (ACCAF) has also worked toward eliminating the practice through regional advocacy [23,24].

In addition to these programmatic efforts, several countries have introduced legislation to combat FGM/C. For instance, Kenya enacted a law criminalizing FGM/C in 2011 [25]. Similarly, the practice has been criminalized in Ethiopia [26] and Tanzania [27]. However, despite these interventions, the prevalence of FGM/C remains high in several parts of East Africa. Socioeconomic and sociocultural factors, particularly low levels of maternal education, have been consistently identified as major contributors to the persistence of the practice [18].

Therefore, this study aims to assess maternal education-related inequality in FGM/C prevalence among daughters aged 0–14 years in three East African countries using DHS data. The findings of this study may provide insights for policymakers and public health planners to consider socioeconomic factors as intervention targets toward the elimination of FGM/C.

## Methods

### Data source

The data used for this analysis were accessed from the DHS archives after submitting a data request with appropriate justification. We used the most recent Demographic and Health Survey data sets available for Ethiopia (2016), Kenya (2022), and Tanzania (2022). These countries were selected based on the availability of information on daughters’ FGM/C status in the Individual Recode (IR) data set. We used the IR and Birth Record (BR) data sets to assess education-related inequality in female genital mutilation/cutting among daughters aged 0–14 years in selected East African countries. We included only respondents who had heard of FGM/C, as the DHS program only collects information on daughters’ FGM/C status from these respondents.

To enable analysis at the daughter level, the IR data set was reorganized using the reshape long command in Stata version 17. This process converted the daughter-specific circumcision variables (g121_x, g122_x, g123_x, and g124_x) and daughters’ birth index variable (gidx_x) from wide to long format such that each row in the data set represented one daughter. The reshaped data set was then merged with the BR data set using the unique identifier (caseid) and daughters’ birth index (gidx). This merge helped to verify the daughters’ ages and birth histories and to restrict the analysis to those aged 0–14 years.

### Study design, setting, and period

**Figure 1.**
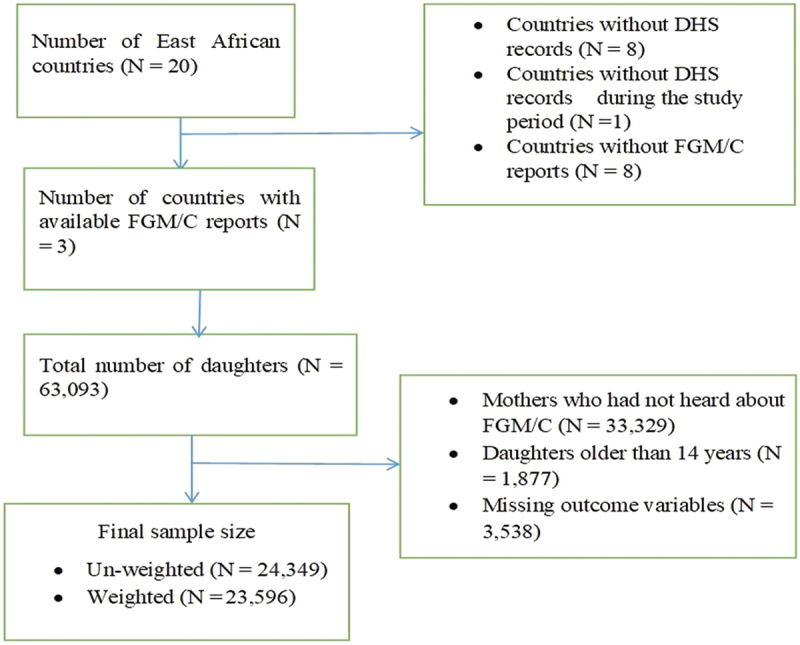
Flow chart of study participant’s selection.

The study used secondary data from Demographic and Health Surveys conducted in three selected East African countries. East Africa was defined according to the United Nations’ geographical classification of the Eastern Africa sub-region, which includes 20 countries [28]. However, only Ethiopia, Kenya, and Tanzania were included because they had recent DHS data sets (2016–2022) containing complete and analyzable information on FGM/C among daughters aged 0–14 years. Other countries in the sub-region were excluded because they did not participate in the DHS program, had not conducted a survey within the study period, or did not include daughter-specific FGM/C data (Figure 1). East Africa is a global hotspot for FGM/C, accounting for approximately one-quarter of all FGM/C survivors worldwide [16].

### Sampling procedures and populations

The source population comprised all daughters aged 0–14 years in East African countries, while the study population included daughters aged 0–14 years residing in selected enumeration areas (EAs) of Ethiopia, Kenya, and Tanzania, based on the availability of FGM/C data in the DHS IR data set between 2016 and 2022. The DHS program employed a stratified two-stage cluster sampling design, with enumeration areas serving as the primary sampling units and households as the secondary sampling units. Detailed information on the sampling procedures is available on the DHS Program website [29].

Sampling weights were applied before analysis to account for the complex survey design and to ensure the representativeness (v005/1,000,000). All analyses were conducted using the survey (svy) command in Stata to adjust for clustering and stratification.

From the IR data set (*N* = 63,093 women), 29,764 having reported having heard of FGM/C. Among these, 3,538 observations were excluded because of missing outcome data. In addition, daughters older than 14 years (*N* = 1,877) were excluded from the analysis. The final weighted sample size consisted of 23,596 daughters aged 0–14 years (Table 1; Figure 1).

**Table 1.** Overall sample size of each country.

country	Year	Unweighted sample size	Weighted sample size
Ethiopia	2016	6,001	6,624
Kenya	2022	12,939	11,660
Tanzania	2022	5,409	5,312
**Total**		**24,349**	**23,596**

### Variables of the study

#### Dependent variable

The dependent variable was the experience of FGM/C among daughters aged 0–14 years, measured as a binary outcome (yes/no) based on maternal reports.

#### Independent variables

Independent variables included maternal FGM/C status, maternal attitude towards the continuation of FGM/C, maternal marital status, maternal age at first cohabitation, household wealth quintile, place of residence, sex of household head, and maternal media exposure.

Inequality in FGM/C among daughters was assessed by maternal educational attainment as ranking variable. Consistent with DHS standards, education was categorized as no education, primary, secondary, or higher [30,31].

Household wealth quintile was classified as poorest, poorer, middle, richer, and richest according to DHS wealth index classification.

Maternal age at first cohabitation was categorized as <18 years and ≥18 years based on the respondent’s self-reported age at first cohabitation.

Media exposure was defined as maternal self-reported access to at least one form of media (radio, television, or newspaper/magazine) at least once per week and was categorized as yes or no.

Maternal attitude towards FGM/C continuation was determined based on the respondents answer whether FGM/C should be continued or discontinued.

### Data management analysis

Data cleaning, coding, and analysis were performed using Stata version 17. The descriptive statistics were presented in tables and text. Maternal education-related inequality in FGM/C was visualized using a concentration curve.

The concentration curve shows the distribution of FGM/C across level of maternal education. It plots the cumulative percentage of FGM/C (y-axis) against the cumulative percentage of mothers, ranked from least to most educated (x-axis). If FGM/C is equally distributed, the curve follows a 45-degree line, known as the line of equality. If FGM/C is more prevalent among the uneducated, the curve lies above the line of equality; if it is more prevalent among the highly educated, it lies below the line of equality [32].

To quantify the degree of inequality, we employed the Erreygers Normalized Concentration Index (ECI). It was computed using Stata’s coindex command. This procedure applies a rank-based approach in which observations are ordered according to the socioeconomic variable (maternal education). Given that maternal education was categorized into four ordered groups (no education, primary, secondary, and higher), tied ranks naturally occurred within categories. In such cases, individuals within the same educational category were assigned identical ranks, consistent with grouped data. The ECI was therefore estimated based on the ranked distribution of the outcome across these ordered educational categories. The ECI is specifically designed for binary variables (0, 1), as the traditional concentration index can be influenced by the mean of binary variables. The Erreygers Concentration Index ranges from −1 to +1. A negative value indicates that FGM/C is more concentrated among daughters of less-educated mothers, while a positive value indicates concentration among daughters of more-educated mothers. A value of zero indicates no inequality [33,34].

The ECI for a health variable y, bounded between 0 and 1, is mathematically expressed as:

$$\mathrm{ECI}=4\mathrm{\mu CI}\left(y\right)$$

Where μ is the mean of binary health variable, y is FGM/C prevalence, $\mathrm{CI}\left(y\right)$ is the traditional concentration index, and ECI is the Erreygers Concentration Index.

### Decomposition analysis

To understand the drivers of this inequality, we decomposed the concentration index. For any linear additive regression model of health outcome y (FGM/C):

$$y=\mu +\sum_{k}\beta_{k}X_{k}+\in$$

The concentration index for y, CI, is computed as:

$$y=\sum_{k}\left(\frac{\beta_{k}{\overset{ˉ}{X}}_{k}}{\mu }\right)C_{k}+\frac{gc_{\in }}{\mu }$$

Where y denotes the health outcome variable (FGM/C), $X_{k}$ is a set of determinants of FGM/C, α is the intercept, $\beta_{k}$ is the coefficient of $X_{k}$, µ is the mean of y, ${\overset{ˉ}{X}}_{k}$ is the mean of $X_{k}$, $C_{k}$ is the CI for $X_{k}$, $gc_{\in }$ is the generalized CI for the error term (∈), $\frac{\beta_{k}{\overset{ˉ}{X}}_{k}}{\mu }$ is the elasticity of y with respect to ${\overset{ˉ}{X}}_{k}$ [34].

The decomposition produced estimates of regression coefficients, elasticities, concentration indices, and the percentage contribution of each determinant to the observed inequality. The coefficients represent the direct association between each determinant and FGM/C, while elasticities capture the responsiveness of FGM/C in daughters to changes in each determinant when moving from one category to the other. The concentration index measures the distribution of each determinant across maternal education levels.

The absolute contribution of each variable to inequality was computed as the product of its elasticity and its concentration index. These contributions can be either positive or negative depending on the joint effect of the elasticity of FGM/C in daughters and the distribution of the determinants across maternal education levels. The percentage contribution shows the relative share of each determinant in explaining the overall maternal education-related inequality in FGM/C. A positive percentage contribution indicates that the variable widens the observed inequality (i.e. is concentrated among lower-educated mothers), while a negative contribution indicates that it narrows the inequality.

### Ethical considerations

This study is based on a secondary data of existing survey collected by the DHS Program (www.measuredhs.com). Data access was granted by the DHS Program data archivists following submission of a research project description. The original DHS surveys were conducted with informed written consent from all participants and were approved by the relevant national ethics committees in the respective countries and by the ICF Institutional Review Board. The study was conducted in accordance with the ethical principles for medical research involving human subjects as outlined in the Declaration of Helsinki.

## Results

### Background characteristics of the study participants

A total of 23,596 weighted daughters aged 0–14 years were included in the study. Characteristics of their mothers and households showed that nearly half of the mothers (47.50%) were aged 25–34 years, 74.46% were married, and 43.21% had attained primary education. The majority of the daughters lived in rural areas (73.10%), and 72.68% resided in male-headed households. About 21.36% of the daughters belonged to households in the poorest wealth quintile. In terms of media exposure, 65.85% of mothers reported exposure to media. Nearly half of the included daughters were from Kenya (49.42%).

The prevalence of FGM/C was higher among daughters whose mothers had no formal education (15.40%) and those from the poorest households (8.02%). Daughters of circumcised mothers had a higher prevalence (37.34%) compared with daughters of uncircumcised mothers. Among the three countries, Ethiopia had the highest prevalence of FGM/C (17.25%), followed by Kenya and Tanzania (Table 2).

**Table 2.** Weighted percentage of FGM/C among daughters aged 0–14 years in three East African countries by background characteristics.

Variables	Categories	FGM/C		Total weighted frequency (%)
		No (*N* = 22,223) 94.18%	Yes (*N* = 1,373) 5.82%	
Daughter’s age	<1 years	1,841 (98.64)	25 (1.36)	1,866 (7.91)
	1–4 years	6,492 (97.53)	158 (2.37)	6,450 (28.18)
	5–9 years	7,625 (94.68)	428 (5.32)	8,053 (34.13)
	10–14 years	6,265 (89.16)	762 (10.84)	7,027 (29.78)
Maternal age	15–24	2,641 (97.64)	63 (2.36)	2,703 (11.46)
	25–34	10,641 (94.95)	566 (5.05)	11,207 (47.50)
	35–49	8,941 (92.32)	744 (7.68)	9,685 (41.04)
Maternal marital status	Single	5,886 (97.68)	140 (2.32)	6,026 (25.54)
	Married	16,337 (92.98)	1,233 (7.02)	17,570 (74.46)
Maternal education	No	5,994 (84.60)	1,091 (15.40)	7,085 (30.03)
	Primary	9,933 (97.42)	263 (2.58)	10,196 (43.21)
	Secondary	4,512 (99.58)	18 (0.42)	4,530 (19.20)
	Higher	1,784 (99.98)	1 (0.02)	1,785 (7.56)
Sex of household	Male	16,085 (93.80)	1,063 (6.20)	17,148 (72.68)
	Female	6,138 (95.19)	310 (4.81)	6,448 (27.32)
Residence	Urban	6,234 (98.20)	114 (1.80)	6,348 (26.90)
	Rural	15,989 (92.7)	1,259 (7.30)	17,248 (73.10)
Wealth Index	Poorest	4,637 (91.98)	404 (8.02)	5,041 (21.36)
	Poor	4,506 (94.36)	270 (5.64)	4,776 (20.24)
	Medium	4,330 (94.17)	268 (5.83)	4,598 (19.49)
	Rich	4,404 (93.24)	319 (6.76)	4,723 (20.02)
	Richest	4,346 (97.49)	112 (2.51)	4,458 (18.90)
FGM/C required by religion	No	20,213 (96.5)	734 (3.5)	20,947 (77.37)
	Yes	2010 (75.86)	639 (24.14)	2,649 (19.47)
Maternal media exposure	Yes	15,184 (97.72)	353 (2.28)	15,537 (65.85)
	No	7,039 (87.35)	1,020 (12.65)	8,059 (34.15)
Maternal FGM/C status	No	14,730 (99.63)	55 (0.37)	14,785 (62.66)
	Yes	7493 (85.04)	1318 (14.96)	8811 (37.34)
Country	Ethiopia	5,482 (82.75)	1,142 (17.25)	6,624 (28.07)
	Kenya	11,463 (98.31)	197 (1.68)	11,660 (49.42)
	Tanzania	5,278 (99.37)	34 (0.63)	5,312 (22.51)

### Prevalence of FGM/C among daughters aged 0–14 years in three selected East African countries

The overall prevalence of FGM/C among daughters aged 0–14 years in the three East African countries was 5.82% (95% CI: 5.53–6.12) (Table 2).

**Figure 2.**
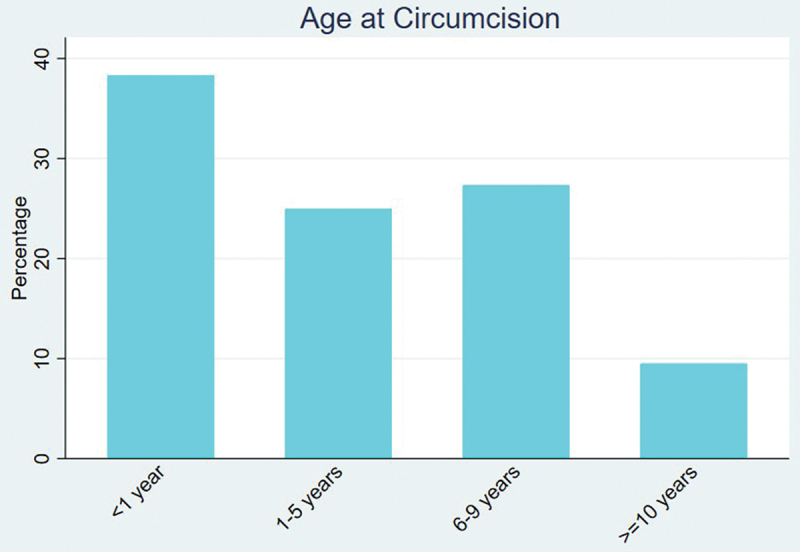
Prevalence of FGM/C among daughters aged 0–14 years across the age category.

Among daughters who had undergone FGM/C, 9.22% had undergone infibulation (sewing of the genital area closed). Most procedures (96.44%) were performed by traditional practitioners, while 3.66% were performed by health-care professionals. Approximately 16.09% of mothers reported that FGM/C is required by their religion, and 10.53% believed the practice should continue. Regarding the age at circumcision, approximately 38.30% of daughters had undergone FGM/C before their first birthday (Figure 2).

### Maternal education-related inequality in female genital mutilation/cutting

**Figure 3.**
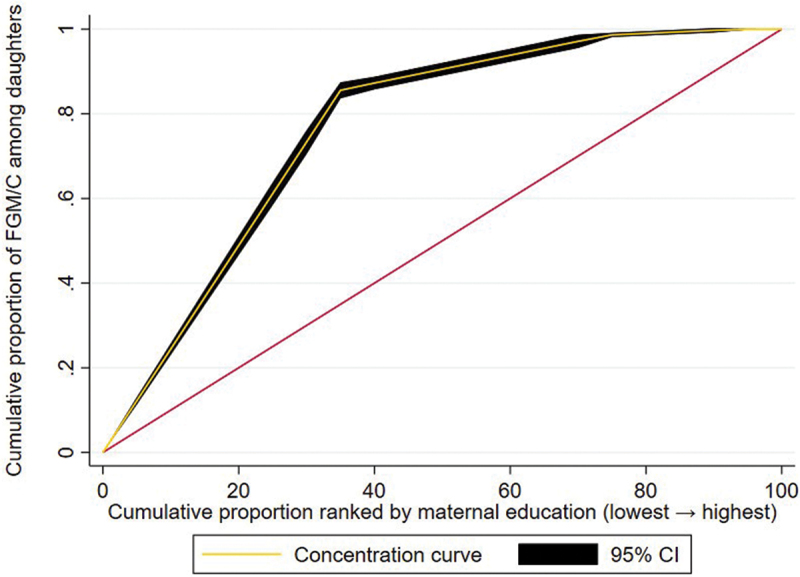
Concentration curve for female genital mutilation/cutting among daughters aged 0–14 years ranked by maternal educational attainment.

The Erreygers normalized concentration index for FGM/C among daughters aged 0–14 years, ranked by maternal educational attainment, was −0.126 (SE = 0.0092, *p* < 0.001), indicating favoring daughters of mothers with lower educational attainment. This indicates that FGM/C was disproportionately concentrated among daughters of mothers with the lowest educational attainment. Consistently, the concentration curve lies above the line of equality, further confirming that FGM/C was more prevalent among daughters whose mothers had lower educational attainment (Figure 3).

### Decomposing the maternal education-related inequality in FGM/C among daughters aged 0–14 years

A decomposition analysis was conducted to examine the contribution of each independent variable to maternal education-related inequality in FGM/C. The analysis provided results including coefficients, elasticities, concentration indices, and the percentage contributions of each variable.

The concentration index represents how the independent variables are distributed across maternal education level. The concentration index can be either positive or negative. A negative value indicates concentration among the less-educated population, while a positive value indicates a concentration among the more-educated. In this study, variables including age 35–49 years, attitude toward continuation of FGM, rural residence, FGM/C being required by religion, maternal FGM/C status, lack of media exposure, poorer and middle wealth status, being married, and age at first cohabitation <18 had negative concentration index values. This indicates that these factors were more prevalent among mothers with lower educational attainment, while female-headed households were concentrated among daughters of more-educated mothers.

The contribution was obtained as the product of elasticity and the concentration index of each variable, and it can take either positive or negative values. A positive percentage contribution indicates that the factor widens the observed maternal education-related inequality, whereas a negative contribution suggests that it narrows the inequality.

For example, maternal FGM/C status had an absolute contribution of −0.03. This reflects the combined contribution of its positive elasticity (elasticity = 0.142) and its concentration among less-educated women (concentration index = −0.210), which implies that it worsens inequality.

The percentage contribution analysis illustrates the relative contribution of each explanatory variable to the observed maternal education-related inequality in FGM/C among daughters. A positive value indicates that a factor contributes to a widening of observed inequality, whereas a negative value indicates that it contributes to a narrowing of inequality.

The findings showed that maternal education accounted for a large share of the observed inequality in FGM/C among daughters (42.51%). Specifically, primary and secondary levels of maternal education had positive contributions, indicating that they contribute to widening inequality among daughters, whereas higher maternal education contributed to narrowing the gap.

Maternal FGM/C status was the second most important contributor (23.79%), indicating that mothers who had undergone FGM/C significantly widened maternal education-related inequality in FGM/C among daughters. Other major contributors included lack of maternal media exposure (22.58%) and rural residence (13.89%), both of which widened inequality. Maternal age at first cohabitation <18 years also contributed positively to the observed inequality. In contrast, household wealth quintiles, particularly the richest group, contributed negatively, thereby narrowing the observed inequality. Collectively, the factors included in the model explained 93.27% of the overall maternal education-related inequality in FGM/C among daughters, with the remaining 6.73% attributed to unexplained residuals (Table 3).

**Table 3.** Contributing factors of maternal education-related inequality in FGM/C among daughters aged 0–14 years.

Variables	Category	Coefficient	Elasticity	Concentration index	Absolute contribution	% Contribution
Age	15–24	Ref				
	25–34	0.02*	0.039	0.046	0.002	−1.46
	35–49	0.047*	0.078	−0.014	−0.001	0.88
	Subtotal				0.001	−0.58
Residence	Urban	Ref				
	Rural	−0.028*	−0.0698	0.407	−0.0284	**13.89**
FGM/C continuation attitude	continued	0.048*	0.02	−0.122	−0.003	1.95
	stopped	Ref				
FGM/C needed by religion	No	Ref				
	Yes	0.15*	0.006	−0.094	−0.001	0.48
Maternal FGM/C status	No	Ref				
	Yes	0.1*	0.142	−0.210	−0.030	**23.79**
Educational level	No education	Ref				
	Primary	0.144*	0.173	−0.291	−0.050	39.94
	Secondary	0.119*	0.205	−0.084	−0.017	13.64
	Higher	0.088*	0.068	0.204	0.013	−11.07
	Subtotal				−0.054	**42.51**
Media exposure	No	−0.026	−0.069	0.406	−0.028	**22.58**
	Yes	Ref				
Age at first marriage	<18	0.026*	0.058	−0.187	−0.010	**8.73**
	>18	Ref				
Household head	Male	Ref				
	Female	0.013*	0.015	−0.025	−0.003	0.3
Wealth index	Poorest	Ref				
	Poorer	0.01	0.007	−0.300	−0.002	1.88
	Middle	0.02	0.015	0.021	0.003	−0.25
	Richer	0.041*	0.033	0.334	0.01	−8.98
	Richest	0.043*	0.033	0.613	0.02	−16.09
	Subtotal				0.031	−23.44
Marital status	Single	Ref				
	Married	0.013*	0.039	−0.034	0.001	1.18
Total						**93.27**

*p value < 0.05.

## Discussion

The findings of this study revealed that FGM/C was disproportionately concentrated among daughters whose mothers had lower levels of education. Previous studies conducted in low- and middle-income countries also showed a positive association between maternal education and FGM/C among daughters. Decomposition analysis also revealed that 42.51% of the maternal education-related inequality in FGM/C among daughters is explained by the direct contribution of maternal education.

Education is key to changing attitudes towards health and increasing awareness of the negative health consequences of FGM/C. In certain communities, FGM/C is seen as a prerequisite for marriage, thus perpetuating the practice. Education can break this cycle by empowering women’s decision-making autonomy, promoting greater investment in their children’s education, and contributing to the gradual change of social norms and attitudes towards FGM/C. Furthermore, education elevates the social status of women by providing opportunities beyond culturally prescribed roles and norms that promote conformity. However, in the absence of education, women are more likely to face strong social and normative pressures reinforcing adherence to FGM/C, thereby contributing to inequalities in its distribution [19,20,35,36].

The prevalence of FGM/C among daughters aged 0–14 years was 5.82% (95% CI: 5.53–6.12). This estimate is lower than previous reports from sub-Saharan Africa, which may be partly explained by differences in study periods [37–39]. This lower prevalence suggests a downward trend in the practice over time. However, although this reduction is encouraging, achieving the elimination of FGM/C by 2030 under the Sustainable Development Goals appears challenging and may be unattainable given the current pace of decline [40]. Although Kenya, Ethiopia, and Tanzania have criminalized FGM/C [25–27], the continued persistence of the practice demonstrates that legislation alone is insufficient to bridge the gap between national policy and local sociocultural realities. The decomposition analysis further indicates that this disparity is largely driven by sociocultural and socioeconomic determinants, particularly maternal education and intergenerational transmission of the practice.

Maternal FGM/C status, media exposure, place of residence, and age at first cohabitation also contributed to the observed inequality, according to the decomposition analysis. The second-largest contributor to the observed inequality, after maternal education, was maternal FGM/C status. Studies show that daughters of circumcised mothers were significantly more likely to undergo FGM/C. This intergenerational transmission contributes to the concentration of FGM/C among daughters of less-educated mothers, thereby reinforcing inequality. These findings may be attributed to the fact that circumcised mothers are more likely to support the continuation of the practice. This support is often rooted in the belief that FGM/C is essential for hygiene, a prerequisite for marriageability, and a means of preserving cultural identity. Furthermore, such decisions are frequently reinforced by religious influences and community pressure [41–45].

Media exposure was another contributing factor to the observed inequality in FGM/C among daughters. Various studies have shown the association between lack of maternal media exposure and an increased likelihood of FGM/C in daughters [46]. Media is a key tool for transmitting important health information, making it essential in campaigns against harmful practices such as FGM/C. Thus, mothers lacking media exposure may miss important information that could potentially protect their daughters from undergoing FGM/C. Consequently, limited media exposure may widen inequality by restricting awareness among already disadvantaged groups [47].

Place of residence was also a contributor to the observed maternal education-related inequality in FGM/C among daughters. Living in a rural area has been highly correlated with FGM/C practice in previous studies [48–50]. In these settings, limited access to formal education, healthcare services, and diverse sources of information may allow traditional beliefs to persist unchallenged and reduce awareness of the human rights implications of the practice. Additionally, stigma, social rejection, and the risk of being undermined by community members for remaining uncircumcised may be more pronounced in rural areas. Collectively, these factors reinforce existing disparities and contribute to the unequal distribution of FGM/C among daughters [51,52].

Maternal age at first cohabitation was also a contributing factor to inequality in FGM/C among daughters. Previous studies have demonstrated an association between FGM/C and child, early, and forced marriage [53,54]. The overlap between child marriage and FGM/C suggests shared cultural and social determinants, which may cluster within the same populations. This clustering can further intensify inequality by increasing the likelihood that daughters of women who married early are disproportionately exposed to FGM/C, thereby reinforcing vulnerability among already disadvantaged groups [53].

## Conclusions

Our study revealed that FGM/C was disproportionately concentrated among daughters whose mothers had lower educational attainment. The observed inequality was largely shaped by maternal and cultural factors, indicating that limited educational attainment and intergenerational transmission of the practice continue to play a substantial role in sustaining FGM/C among daughters. These findings suggest that strategies aimed at eliminating FGM/C should extend beyond legal enforcement and emphasize improving women’s educational attainment and addressing factors that perpetuate the practice, especially among disadvantaged and rural populations.

The major strength of this study is the use of large-scale, nationally representative DHS data, which enhances the generalizability of the findings. However, several limitations should be considered. Information on FGM/C status among daughters was based on maternal reports, which may be subject to social desirability and recall bias. The inclusion of only three East African countries (due to data availability) limits the generalizability of the findings to the entire region and to other high-prevalence settings with different cultural contexts. In addition, the use of a categorical ranking variable rather than a continuous measure (years of schooling) means that within-group variation in educational attainment is not captured; as a result, the estimates may represent a lower-bound of inequality. Therefore, the concentration index should be interpreted as conservative estimates of the underlying inequality.

## Data Availability

The data can be accessed from the DHS website (www.dhsprogram.com).

## Abbreviations

ACCAF: Africa Coordinating Center for the Abandonment of Female Genital Mutilation
DHS: Demographic and Health Survey
EAs: Enumeration Areas
ECI: Erreygers Concentration Index
FGM/C: Female Genital Mutilation/Cutting
SDG: Sustainable Development Goal
UNICEF: United Nations International Children’s Emergency Fund
UNFPA: United Nations Population Fund
WHO: World Health Organization
